# Double Fracture of the Inferior Maxilla

**Published:** 1866-01

**Authors:** J. L. Wylie

**Affiliations:** Ripley, O.


### Selections.

### DOUBLE FRACTURE OF THE INFERIOR MAXILLA

BY J. L. WYLIE, M.D. RIPLEY, O.

On the 19th of June, 1865, I was hastily called to visit a gentleman some six miles distant, who had been thrown from a loaded flour wagon, the hind wheel having passed over the Inferior Maxilla. Upon my arrival I found him entirely conscious, but laboring from the violence of the shock which his system had sustained. An examination of the jaw revealed a fracture of each side at the junction of the body and ramus compound and comminuted upon the right, transverse and compound upon the left. There was great contusion of the right shoulder and side of the neck as well as of the soft parts of the chin, defining the course of the wheel; the treatment adopted was as follows: Splints of paste-board were moistened in water, enveloped in cotton and moulded to the jaw of each side; the superior extremity pressing firmly against the glenoid process, the inferior extremity reaching the mid-point of the body. A four tailed bandage was used for the purpose of supporting the jaw as well as for maintaining the splints *in situ*. In addition to this a sub-mental bandage was used for the purpose of giving support to the jaw as well as to keep the anterior surfaces of the fractures sufficiently depressed. Owing to the anatomical conformation of the parts, there was in this as in similar fractures, but little displacement. The chief barrier to a successful issue consisted in maintaining the position of the parts. This was without great difficulty effected by means of the simple dressings used without resorting to the more cumbrous and uncomfortable dressings frequently had recourse to in maxillary fractures.

In the course of twenty-four hours from the adjustment of the fracture great swelling and congestion of the soft parts supervened, in consequence of the fracture itself as well as of the contusion effected by the wheel. Owing to this condition the patient was entirely unable to swallow fluids of any kind, and asphyxia would be inevitable if the swelling could not be alleviated. At this juncture a large blister (emp. canth.) was applied to the posterior surface of the neck with the effect of alleviating the swelling. Notwithstanding the patient was robust and plethoric venesection was not resorted to for the reason that union of the right if not of the left side would not probably be effected without suppuration on the account of the comminution, and that owing to that surmised condition, the treatment of nurturing his energy would be more plausible than that of combating an imaginary *sthenia.* As was surmised, suppuration of both sides occurred without any detachment of osseous spiculæ. The pus, constantly of a laudable nature, escaped externally, the wounds kindly healed, the patient speedily gained health and the jaw is now in *statu quo.* Subsequently to the union of the fractures, a swelling occurred upon the right side of the neck, and despite the most energetic revulsive treatment, suppuration occurred, which was indicated by chills, fever, etc., the usual concomitants of extensive suppuration. It will be proper to remark *en passant*, that this effusion, swellings, etc., were the results of the contusion at the time of the accident and related by no continuity with the maxillary fractures. On account of the depth of the pus, the *positive* evidences of suppuration were not discernible, and with the view of revulsion if pus did not exist or with the view of promoting suppuration if it did exist, a blister was applied over the swelling with the effect of transposing the presumptive symptoms for those that were positive. A free opening gave exit to an abundant quantity of offensive pus. After its evacuation, adynamic symptoms speedily gave way to those of convalescence. Owing to the removal of the patient without the circuit of my practice, I was kindly and ably assisted in the after-treatment by Dr. W. A. Dixon.

There are, undoubtedly, points of interest attaching to this case. In the first place, the extent of the fractures; Secondly, the complications from the contusion of adjoining structures; Thirdly, the complete union of the compound comminuted fracture without the detatchment of bone; Fourthly, the precise preservation of the symmetry of the parts, and we might add; Fifthly, the rarity of similar cases in civil fractures.—*Cin. Lancet and Obs.*

